# Insulin sensitivity and mitochondrial homeostasis connect lactation to maternal metabolic health

**DOI:** 10.1172/JCI210439

**Published:** 2026-10-15

**Authors:** Laura L. Hernandez

**Affiliations:** Department of Animal and Dairy Sciences, Affiliate Department of Obstetrics and Gynecology, University of Wisconsin-Madison, Madison, Wisconsin, USA.

## Abstract

Lactation insufficiency in postpartum women has been associated with obesity and insulin resistance, among other risk factors. Challenges related to translating findings from animal models of lactation and the limited ability to study pregnant and lactating women in clinical trials have impeded more detailed mechanistic understanding of this relationship. In this issue of the *JCI*, Meng et al. combined studies in human milk samples and in vitro mammary epithelial cell models from humans and rodents to provide compelling evidence that mitochondrial dysfunction mediates the link between insulin resistance and lactation insufficiency. Notably, they observed that chronic exposure to high levels of insulin led to mitochondrial fragmentation and loss of volume in mouse mammary epithelial cells. This framework linking mitochondria, insulin signaling, and lactation should be extended to in vivo models and primary human tissues to better define the mechanisms supporting successful lactation.

## Introduction

Lactation onset is triggered by hormonal fluctuations resulting from delivery of the placenta and is accompanied by profound upregulation of gene expression in mammary epithelial cells, which supports increased mammary synthetic capacity. Delayed onset of lactation increases the risk for lactation insufficiency, which is associated with low infant weight gain and factors into the inability to breastfeed or decision to stop breastfeeding. The inability to breastfeed has clinically significant consequences for both mother and offspring: it impacts women both emotionally and psychologically, and it prevents babies from accessing the benefits of breast milk.

## Insufficient glandular tissue and low milk supply

An estimated 5%–15% of women are unable to produce sufficient breast milk because of inadequate mammary gland development, a condition referred to as “insufficient glandular tissue,” or IGT (1). Early studies describing IGT were limited by a lack of direct assessment of milk production and were conducted in fairly homogenous, healthy patient populations (2). Some of the suspected risk factors for IGT include maternal history of infertility, polycystic ovary syndrome, hypertension, thyroid dysfunction, hyperandrogenism, advanced maternal age (AMA; >35 years of age or older), pre-pregnancy obesity, preeclampsia, and breast reduction (3, 4); however, some women experience IGT based on anatomic deficiency without any of the above risk factors (5). Studies using human mammary epithelial cells have demonstrated that insulin signaling regulates mammosphere formation and milk protein gene expression (6), raising the possibility that impaired insulin sensitivity contributes to mammary gland and lactational dysfunction. Indeed, emerging evidence suggests that insulin resistance may play an important role in both IGT and low milk supply (LMS) (7).

Unlike IGT, which reflects inadequate mammary gland development, LMS is a multifactorial clinical condition that may arise from numerous maternal or infant factors. In a recent study of 460 patients in Australia, LMS was reported in a startling 38% of women over a 24-hour measured period of milk production (8). Maternal obesity and AMA have been consistently identified as important risk factors. In a cohort of 609 women, both a BMI greater than 25 and AMA were associated with LMS, while women older than 40 years diagnosed with overweight or obesity experienced a 44% greater incidence of delayed lactogenesis (onset later than 72 hours postpartum) (9). Similarly, women with gestational diabetes have been reported to experience delayed initiation of breastfeeding and LMS (10), further supporting a role for metabolic dysfunction in impaired lactation.

The consequences for LMS extend beyond maternal breastfeeding success. Infants born to women with perceived LMS, based on self-reported 24-hour milk production, exhibit reduced weight-for-length gain during the first four months of life (11). Moreover, women with severe LMS have higher rates of obesity, elevated circulating and milk concentrations of TGF-α, and reduced concentrations of long-chain fatty acids in breast milk (12), suggesting that maternal metabolic health influences both milk quantity and composition.

Despite growing evidence linking obesity and insulin resistance with impaired lactation, the underlying biological mechanisms remain poorly understood. Lactation physiology in general has historically been an understudied area of female and reproductive biology. Although substantially more research has been performed in animal models, comparatively few controlled studies have been performed in women, often due to categorization of pregnant and lactating women as protected participants. Elucidating the mechanisms underlying human mammary gland physiology is challenging because it requires the collection of sensitive biological samples (e.g., breast milk, mammary tissue), and prospective or randomized controlled studies are often difficult to conduct in breastfeeding women. Furthermore, the mechanisms underpinning lactation physiology in various animal models, while informative and conducted under highly controlled conditions, are not fully transferable to humans.

This gap in mechanistic understanding is further highlighted by intervention studies targeting maternal metabolism. For example, treatment with metformin improved milk production in only 20% of women with insulin resistance and LMS (13), indicating that insulin resistance alone cannot explain lactational insufficiency. Collectively, these results suggest that metabolic dysfunction contributes to impaired lactation but that additional mechanisms regulating mammary gland development and function remain to be identified.

In this issue of the *JCI*, Meng et al. examined the role of insulin resistance in regulating lactation insufficiency in humans using a combination of human milk samples and in vitro mammary epithelial cell models from humans and rodents (14). To date, due to the limitations discussed above, researchers have yet to delineate the biology underpinning IGT and LMS. The work herein highlights the need for mechanistic studies in prospectively controlled animal and in vitro models to determine the true etiologies of IGT and LMS, to better support women affected by these conditions.

## Insulin resistance and milk production

Meng et al. provided compelling evidence that insulin signaling is a critical regulator of human milk production and identified mitochondrial dysfunction as a potential mechanistic link between maternal insulin resistance and impaired lactation (14). Because fasting blood samples are difficult to obtain during late pregnancy, human participants were categorized based on their plasma leptin/adiponectin ratio (LAR) at 36 weeks of gestation as a measure of insulin resistance. Women with higher LAR exhibited delayed onset of lactogenesis as measured by increased milk sodium and decreased lactose concentrations. Integrating analyses of human milk samples with mechanistic studies in human and rodent mammary epithelial cell models, the authors demonstrated that insulin regulates mammary epithelial cell function through the AKT signaling pathway and identified widespread alterations in mitochondrial metabolism that were associated with insulin resistance (Figure 1A).

A notable strength of the study is its use of bulk RNA sequencing of milk fat globules (MFGs), which are a source for studying gene expression in mammary epithelial cells (15, 16). This approach enabled the authors to define transcriptional signatures associated with maternal insulin resistance and subsequently leverage these data to conduct mechanistic studies in mammary epithelial cell cultures. While this approach is informative and practical, MFG data are not always fully representative of the functional lactating mammary gland, which comprises a complex set of cell types, as observed in bovine, hamster, and mouse models (17, 18). To date, studies have not confirmed the transcriptomic signatures of MFGs and breast tissue during lactation in women; the possible interactions of other cell types on LMS and IGT have yet to be explored.

To establish causality, the authors complemented their human MFG studies with experiments in mammary epithelial cell lines (14). Using human MCF-12A cells, an immortalized cell line representative of luminal epithelial cells, they demonstrated that AKT signaling is a major regulator of oxidative phosphorylation, supporting a central role for insulin in maintaining mitochondrial function in mammary tissue. While these findings suggest that AKT is critical for regulating insulin’s action, a limitation of the experiments is that they do not examine these findings under differentiated, lactogenic conditions, which are more representative of the biological time frame of interest. The authors extended their RNA-level observations in the MCF12A cell line and milk MFG samples by varying insulin concentrations in the mouse mammary epithelial cell line HC11. They observed increases in extracellular acidification, glycolytic capacity, and oxygen consumption, as measured by cell respiration assays. Notably, exposure to high insulin concentrations induced mitochondrial fragmentation and reduced mitochondrial volume, providing additional evidence that chronic insulin exposure alters mitochondrial homeostasis in mammary cells (Figure 1B). Future work should examine these findings in a human mammary epithelial cell model and possibly even primary human mammary epithelial cells to ensure repeatability of the findings from the mouse cell line.

Taken together, Meng et al.’s work identifies insulin signaling and mitochondrial metabolism as central regulators of mammary epithelial cell function and provides an important mechanistic framework linking maternal metabolic health and lactation capacity (Figure 1C). Caution is warranted when applying the hypothesis that insulin resistance is the primary cause of LMS and/or IGT, as the mammary gland comprises a heterogeneous cell population and numerous other cell types that may play crucial roles. Future studies incorporating in vivo models, primary human tissues, and emerging spatial and single-cell approaches will be essential to define how insulin signaling integrates with the broader cellular ecosystem of the mammary gland to regulate successful lactation.

## Conflict of interest

The author has declared that no conflict of interest exists.

## Figures and Tables

**Figure 1:**
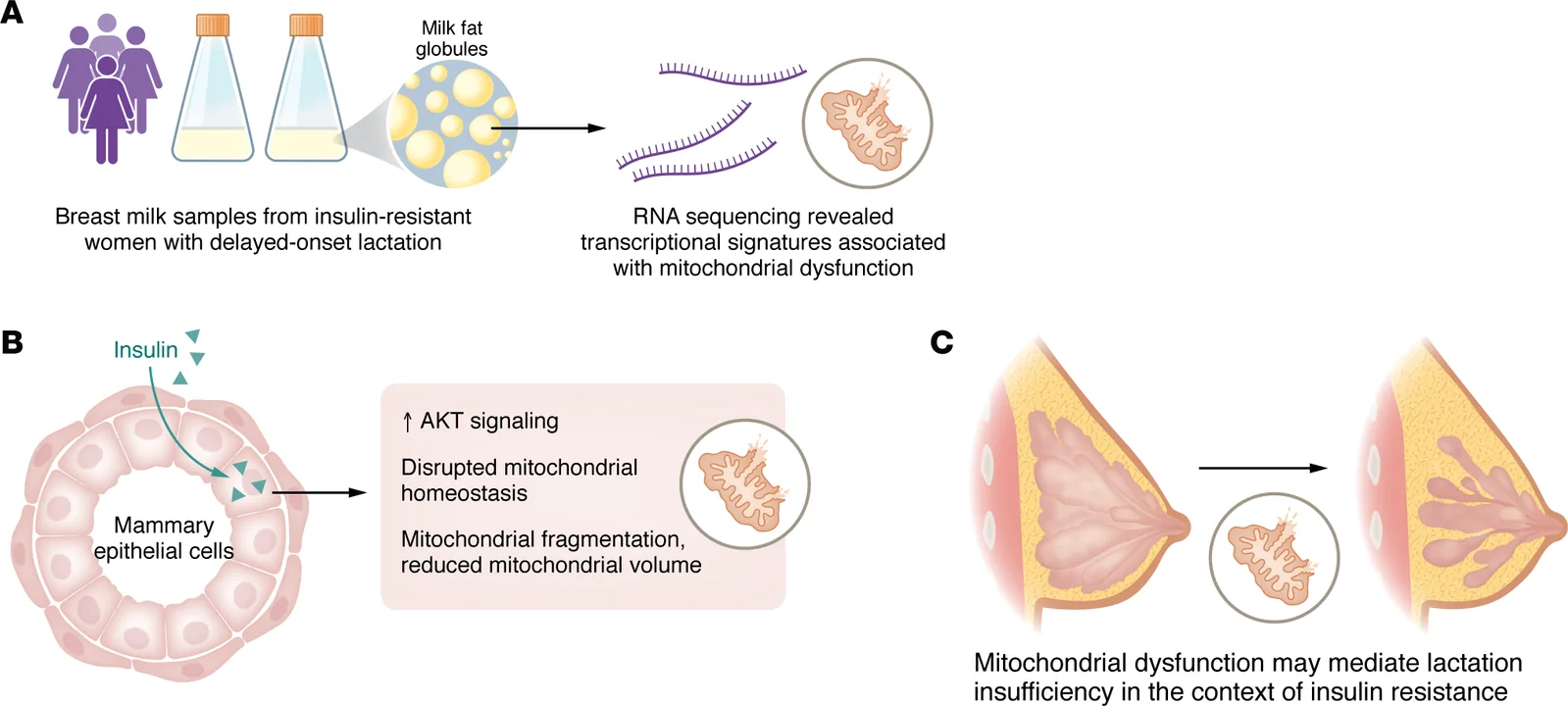
Investigating the association between insulin resistance and insufficient glandular tissue and low milk supply. (**A**) Meng et al. (14) used RNA extraction of the milk fat globule to look at gene expression in mammary epithelial cells in pregnant women who were categorized as insulin resistant at 36 weeks’ gestation. Women with increased insulin resistance exhibited increased expression of pathways related to mitochondrial dysfunction compared with insulin-sensitive participants. (**B**) In human and mouse mammary epithelial cells, treatment with high concentrations of insulin increased AKT signaling. The authors also observed increases in oxidative phosphorylation, extracellular acidification, glycolytic capacity, and oxygen consumption consistent with disruptions in mitochondrial homeostasis as well as a reduction in mitochondrial volume and increased mitochondrial fragmentation. (**C**) Together, the findings indicate that mitochondrial dysfunction links insulin resistance to lactation insufficiency.

## Version 1 10/15/2026

Electronic publication
